# Supplementary material for: Development and characterization of patient-derived xenografts from non-small cell lung cancer brain metastases
Source: Sci Rep. 2021 Jan 28;11:2520. doi: 10.1038/s41598-021-81832-1 (PMC7843608; doi:10.1038/s41598-021-81832-1)
Supplement: Supplementary file 1 — Supplementary Tables 1–3. [file 41598_2021_81832_MOESM1_ESM.pdf]

Development and Characterization of Patient-Derived Xenografts from Non-Small Cell Lung Cancer Brain Metastases

Andrew M. Baschnagel, M.D.,[1,2] Saakshi Kaushik, M.S.,[1] Arda Durmaz, M.S.,[3] Steve Goldstein, Ph.D.,[2,4] Irene M. Ong, Ph.D.,[2,4,5] Lindsey Abel, B.S.,[1] Paul A. Clark, Ph.D.,[1] Zafer Gurel, Ph.D.[1] Ticiana Leal, M.D.,[2.6] Darya Buehler, M.D.,[2,7] Gopal Iyer, Ph.D.,[1,2] Jacob G. Scott, M.D., D.Phil.,[8] Randall J. Kimple, M.D., Ph.D.[1,2]

1. Department of Human Oncology, School of Medicine and Public Health, University of Wisconsin, Madison, WI, USA
2. University of Wisconsin Carbone Cancer Center, School of Medicine and Public Health, University of Wisconsin, Madison, WI, USA
3. Case Western Reserve University, Cleveland, OH 44195, USA
4. Department of Biostatistics and Medical Informatics, University of Wisconsin School of Medicine and Public Health, University of Wisconsin, Madison, WI, USA
5. Department of Obstetrics and Gynecology, University of Wisconsin School of Medicine and Public Health, University of Wisconsin, Madison, WI, USA
6. Department of Medicine, Division of Hematology/Oncology, School of Medicine and Public Health, University of Wisconsin, Madison, WI, USA
7. Department of Pathology and Laboratory Medicine, University of Wisconsin School of Medicine and Public Health, University of Wisconsin, Madison, WI, USA
8. Department of Radiation Oncology, Taussig Cancer Institute, Cleveland Clinic, 10201 Carnegie Ave, Cleveland, OH 44195, USA

Supplemental Materials

Supplemental Methods: Immunohistochemistry Antibodies used in this study

Supplemental Table 1: Detailed molecular profile of brain metastases

Supplemental Table 2: Short tandem repeat analysis

Supplemental Table 3: KEGG (Kyoto Encyclopedia of Genes and Genomes) Pathways

Supplemental Table 4: Gene Ontology Terms

Supplemental Methods

Immunohistochemistry antibodies used in this study

Antibody	Source	Company	Catalog no.	Dilutions
TTF-1 (SPT24)	Mouse	BioCare	ACI3126C	1:100
Ki-67 (D2H10)	Rabbit	Cell Signaling	9027	1:400
phospho-p44/42 MAPK (Erk1/2) (Thr202/Tyr204)	Rabbit	Cell Signaling Technology	4370	1:200
phospho-S6 Ribosomal Protein (Ser235/236)	Rabbit	Cell Signaling Technology	4858	1:400
MET (D1C2)	Rabbit	Cell Signaling Technology	8198	1:320
Phospho-Met (Tyr1234/1235)	Rabbit	Cell Signaling Technology	3077	1:320

Supplemental Table 1: Detailed molecular profile of brain metastases

PDX#	PDX Success	Histology	PD-L1 expression	Tumor Mutation Burden	Mutation Profile
**UW-lung-1**	no	adenocarcinoma	<1%		ERBB2 A775_G776insYVMA amplification- equivocal<br>AURKA amplification- equivocal<br>NFKBIA amplification<br>NKX2-1 amplification<br>ZNF217 amplification
**UW-lung-2**	yes	adenocarcinoma	70%	19 Muts/Mb	KRAS G12C<br>TP53 splice site 994-2A>T<br>CDKN2A p16INK4a E119* p14ARF *133Lext*23<br>RBM10 splice site 1575+1G>C<br>SMARCA4 1548G>C, 2735_2736delGCinsTG<br>ATM 4626G>C<br>KEAP1 599A>C<br>NFKBIA amplification<br>NKX2-1 amplification<br>MYC amplification
**UW-lung-3**	yes	adenocarcinoma	20%		NOTCH4 1099G>T<br>PLCG2 3616C>T<br>KMT2D 10993C>G
**UW-lung-4**	yes	adenocarcinoma	15%	25 Muts/Mb	KRAS G12V 35G>T<br>ATM splice variant 3285-2A>T<br>FBXW7 337_339delGAG<br>NFE2L2 101G>A<br>RBM10 1285C>T<br>SF3B1 1998G>T<br>GNAQ 943G>T<br>JAK2 1993G>C<br>LRP1B 10532-16_10532-15insT, 2966C>A<br>PIK3C2B 260C>G<br>KMT2D 9557G>T
**UW-lung-5**	yes	adenocarcinoma	<1%	9 Muts/Mb	KRAS G12C 34G>T<br>STK11 splice site 465-2A>T<br>CDKN2A loss<br>IGF1R amplification – equivocal<br>KEAP1 splice site 1708+1G>T
**UW-lung-6**	no	adenocarcinoma	<1%	high	TP53 W146fs*3<br>EGFR amplification<br>RICTOR amplification<br>ARID2 P1377fs*9<br>BCORL1 splice site 4697-2A>G<br>DNMT3A truncation intron 22<br>FGF10 amplification<br>GATA1 S30
**UW-lung-7**	yes	adenocarcinoma	50%		TP53 379T>C, 718A>G – subclonal<br>DNMT3A 2478+13G>A<br>KMT2D 4021-15_4021-14delCT

					AKT2 amplification<br>CCNE1 amplification
**UW-lung-8**	no	adenocarcinoma	2%	11 Muts/Mb	KRAS G12V<br>STK11 M129fs*32<br>TP53 splice site 375G>T<br>CRKL amplification<br>EP300 A1629V<br>GATA4 loss exons 3-7<br>NFKBIA amplification<br>NKX2-1 amplification
**UW-lung-9**	no	adenocarcinoma	<1%		NOTCH4 1099G>T<br>PLCG2 3616C>T<br>KMT2D 0993C>G
**UW-lung-10**	no	adenocarcinoma			TP53 2049C>T<br>KMT2D c.4003delT<br>AKT3 c.819+1G>A<br>PDGFRA c.2339delA
**UW-lung-16**	yes	adenocarcinoma	<1%		KRAS G12C 34g>T<br>TP53 R213* 637C>T<br>STK11 40delG frameshift<br>CDKN2A 322G>A<br>APC 1240C>T<br>CHEK2 1556C>T<br>MTOR 3676G>C, 1198G>A<br>PIK3CA 733G>C<br>KEAP1 679C>T stop gained
**UW-lung-18**	yes	adenocarcinoma	35%		KRAS G13C 37G>T<br>TP53 C135Y 404G>A<br>KDR 3872G>T, 2516C>A<br>RAD54L 604C>T<br>KEAP1 1435G>T
**UW-lung-20**	yes	sarcomatoid carcinoma with adenocarcinoma component	100%	9.2 Muts/Mb	TP53 c.376-2A>t Splice region Variant - LOF<br>TP53 p.R2495 Missense variant-LOF<br>CKS1B copy number gain<br>CDKN2A 206A>G<br>BCL6 729_731dupCAG<br>ARID2 3151C>T, 3337G>T<br>HGF 685G>T<br>MET Asp1000 frameshift
**UW-lung-21**	yes	adenocarcinoma	100%		MET exon 14 deletion<br>MET exon 14 splice site mutation<br>TP53 1025G>C<br>CCND1 amp 7<br>CDKN2A 206A>G<br>TERT 228C>T

PD-L1, programmed death-ligand 1; PDX, patient-derived xenograft; UW, University of Wisconsin

Supplemental Table 2: Short tandem repeat analysis

	UW-lung-2					UW-lung-3			UW-lung-4			UW-lung-5		
Sites tested	P0	P1	P5	Brain	Cells P9	P0	P1	P5	P0	P1	P5	P0	P2	P5
D3S1358	16	16	16	16	16	16, 17	17	17	15	15	15	14, 15	14	14
D21S11	29, 32.2	29, 32.2	29, 32.2	29, 32.2	29, 32.2	28, 31.2	28, 31.2	28, 31.2	28, 29	28, 29	28, 29	30, 31.2	31.2	31.2
D18S51	16	16	16	16	16	13	13	13	12, 17	12, 17	12, 17	17, 20	20	20
Penta E	7, 12	7	7	7	7	10, 12	9, 10	9, 10	5, 15	5, 15	5, 15	7, 10	7, 10	7, 10
Penta D	13, 14	13, 14	13, 14	13, 14	13, 14	11, 12	11, 12	11, 12	9, 11	9, 11	9, 11	9, 10	9	9
D8S1179	10, 13	10, 13	10, 13	10, 13	10, 13	12, 13, 14	13, 14	13, 14	10, 12	10, 12	10, 12	13, 14	13, 14	13, 14
FGA	22, 24	22, 24	22, 24	22, 24	22, 24	22, 23, 24	22, 23	22, 23	23, 25	23, 25	23, 25	21, 23	23	23
D5S818	11, 12	11	11	11	11	11	11	11	11	11	11	11, 13	11	11
D13S317	11, 12	11, 12	11, 12	11, 12	11, 12	8, 9	9	9	9, 12	9, 12	9, 12	11, 12	11, 12	11, 12
D7S820	7, 12	7, 12	7, 12	7, 12	7, 12	10, 11	11	11	10, 12	10, 12	10, 12	8, 11	8, 11	8, 11
D16S539	11, 12	12	12	12	12	9, 10	10	10	9, 11	9, 11	9, 11	11, 12	11	11
vWA	17, 18	17	17	17	17	14, 17	14	14	16	16	16, 17	16, 19	19	19
THO1	9, 9.3	9, 9.3	9, 9.3	9, 9.3	9, 9.3	9, 9.3	9, 9.3	9, 9.3	9, 9.3	9, 9.3	9, 9.3	7, 9.3	7, 9.3	7, 9.3
Amelogenin	X	X	X	X	X	X, Y	X	X	X, Y	X, Y	X, Y	X	X	X
TPOX	8	8	8	8	8	11, 12	11, 12	11, 12	8, 10	8, 10	8, 10	10, 11	10, 11	10, 11
CSF1PO	10, 11	10	10	10	10	11, 12	11, 12	11, 12	11, 12	11, 12	11, 12	12, 15	12	12
Percent Match		100	100	100	100		91.7	91.7		95.4	91.3		100	100

	UW-lung-7			UW-lung-16					UW-lung-18			UW-lung-20			UW-lung-21		
Sites tested	P0	P2	P5	P0	P1	P5	Brain	Cells P15	P0	P1	P5	P0	P1	P5	P0	P1	P5
D3S1358	16, 17	16, 17	16, 17	17	17	16, 17	15, 17	15, 17	15, 16	15	15	17, 18	17, 18	17, 18	16	16	16
D21S11	28, 31.2	31.2	31.2	29, 30	30	29, 30, 32.2	30	30, 32.2	30, 32.2	30, 32.2	32.2	28, 33.2	28, 33.2	28, 33.2	29, 30	29, 30	29, 30
D18S51	13	13	13	12	12	12, 16	12, 13	12, 13	13	13	13	13, 17	13, 17	17	15, 21	21	21
Penta E	5, 13	5, 13	5, 13	13, 16	13, 16	7, 13, 16	13, 16	7, 11, 13, 16	7, 11	7, 11	7, 11	7, 16	7, 16	7, 16	8, 15	8, 15	8, 15
Penta D	11, 12	11	11	8, 12	8, 12	8, 12, 13, 14	8, 12	8, 12	12	12	12	10, 13	10, 13	10, 13	12, 14	12, 14	12, 14
D8S1179	12, 15	12, 15, 16	12, 15, 16	12, 14	12, 14	10, 12, 13, 14	12, 14	12, 14	12, 14	12, 14	12, 14	11, 13	11, 13	11, 13	12, 16	12, 16	12, 16
FGA	19, 23	19, 20, 22	19, 20, 22	21, 26	21, 26	21, 22, 24, 26	21, 26	21, 26	21, 22	21	21	20	20	20	21, 21.2	21, 21.2	21, 21.2
D5S818	11, 12	11, 12	11, 12	12, 13	12, 13	11, 12, 13	12, 13	12, 13	13	13	13	12	12	12	11, 12	11	11
D13S317	10, 11	10	10, 11	12, 13	12, 13	11, 12, 13	12, 13	11, 12, 13	11, 12	11, 12	11, 12	11, 12	11, 12	11	10	10	10
D7S820	9, 11	9, 11	9, 11	8, 10	8, 10	7, 8, 10, 12	8, 10	8, 10, 11	10, 11	10, 11	11	10, 12	10, 12	10, 12	10, 11	11	11
D16S539	11, 12	11, 12	11, 12	11	11	11, 12	11	11, 12	12	12	12	9, 13	9, 13	13	12	12	12
vWA	17, 18	18	18	17	17	17	17, 18	17, 18	15, 18	15, 18	18	16, 17	16, 17	16	14, 16	14, 16	14, 16
THO1	7, 8	7, 8	7, 8	9.3	9.3	9, 9.3	9.3	9, 9.3	8, 9	9	9	8, 9	8, 9	8, 9	9, 9.3	9, 9.3	9, 9.3
Amelogenin	X	X	X	X, Y	X, Y	X, Y	X, Y	X, Y	X	X	X	X	X	X	X, Y	X, Y	X, Y
TPOX	8	8	8	8	8	8	8	8, 9	8, 9	8, 9	8, 9	8	8	8	8, 9	8, 9	8, 9
CSF1PO	11, 12	11, 12	11, 12	11, 13	11, 13	10, 11, 13	11, 13	9, 11, 13	9, 12, 13	9, 12, 13	9, 13	10, 12	10, 12	10	10, 12	10	10
Percent Match		89	89		100	60	92.8	81		100	100		100	100		100	100

Supplemental Table 3: KEGG (Kyoto Encyclopedia of Genes and Genomes) Pathways

qValue positive log fold change

geneset	URL	size	qValue	direction
KEGG_ALLOGRAFT_REJECTION	http://software.broadinstitute.org/gsea/msigdb/cards/KEGG_ALLOGRAFT_REJECTION	27	0	DOWN
KEGG_ASTHMA	http://software.broadinstitute.org/gsea/msigdb/cards/KEGG_ASTHMA	15	0	DOWN
KEGG_AUTOIMMUNE_THYROID_DISEASE	http://software.broadinstitute.org/gsea/msigdb/cards/KEGG_AUTOIMMUNE_THYROID_DISEASE	29	0	DOWN
KEGG_CELL_ADHESION_MOLECULES_CAMS	http://software.broadinstitute.org/gsea/msigdb/cards/KEGG_CELL_ADHESION_MOLECULES_CAMS	122	0	DOWN
KEGG_CHEMOKINE_SIGNALING_PATHWAY	http://software.broadinstitute.org/gsea/msigdb/cards/KEGG_CHEMOKINE_SIGNALING_PATHWAY	162	0	DOWN
KEGG_COMPLEMENT_AND_COAGULATION_CASCADES	http://software.broadinstitute.org/gsea/msigdb/cards/KEGG_COMPLEMENT_AND_COAGULATION_CASCADES	53	0	DOWN
KEGG_CYTOKINE_CYTOKINE_RECEPTOR_INTERACTION	http://software.broadinstitute.org/gsea/msigdb/cards/KEGG_CYTOKINE_CYTOKINE_RECEPTOR_INTERACTION	185	0	DOWN
KEGG_ECM_RECEPTOR_INTERACTION	http://software.broadinstitute.org/gsea/msigdb/cards/KEGG_ECM_RECEPTOR_INTERACTION	77	0	DOWN
KEGG_GRAFT_VERSUS_HOST_DISEASE	http://software.broadinstitute.org/gsea/msigdb/cards/KEGG_GRAFT_VERSUS_HOST_DISEASE	30	0	DOWN
KEGG_HEMATOPOIETIC_CELL_LINEAGE	http://software.broadinstitute.org/gsea/msigdb/cards/KEGG_HEMATOPOIETIC_CELL_LINEAGE	66	0	DOWN
KEGG_INTESTINAL_IMMUNE_NETWORK_FOR_IGA_PRODUCTION	http://software.broadinstitute.org/gsea/msigdb/cards/KEGG_INTESTINAL_IMMUNE_NETWORK_FOR_IGA_PRODUCTION	34	0	DOWN
KEGG_LEISHMANIA_INFECTION	http://software.broadinstitute.org/gsea/msigdb/cards/KEGG_LEISHMANIA_INFECTION	66	0	DOWN
KEGG_NEUROACTIVE_LIGAND_RECEPTOR_INTERACTION	http://software.broadinstitute.org/gsea/msigdb/cards/KEGG_NEUROACTIVE_LIGAND_RECEPTOR_INTERACTION	148	0	DOWN
KEGG_TYPE_I_DIABETES_MELLITUS	http://software.broadinstitute.org/gsea/msigdb/cards/KEGG_TYPE_I_DIABETES_MELLITUS	34	0	DOWN
KEGG_VIRAL_MYOCARDITIS	http://software.broadinstitute.org/gsea/msigdb/cards/KEGG_VIRAL_MYOCARDITIS	58	0	DOWN
KEGG_FOCAL_ADHESION	http://software.broadinstitute.org/gsea/msigdb/cards/KEGG_FOCAL_ADHESION	185	8.27E-05	DOWN
KEGG_LEUKOCYTE_TRANSENDOTHELIAL_MIGRATION	http://software.broadinstitute.org/gsea/msigdb/cards/KEGG_LEUKOCYTE_TRANSENDOTHELIAL_MIGRATION	104	8.79E-05	DOWN
KEGG_PRIMARY_IMMUNODEFICIENCY	http://software.broadinstitute.org/gsea/msigdb/cards/KEGG_PRIMARY_IMMUNODEFICIENCY	28	1.45E-04	DOWN
KEGG_ANTIGEN_PROCESSING_AND_PRESENTATION	http://software.broadinstitute.org/gsea/msigdb/cards/KEGG_ANTIGEN_PROCESSING_AND_PRESENTATION	57	1.54E-04	DOWN
KEGG_CALCIUM_SIGNALING_PATHWAY	http://software.broadinstitute.org/gsea/msigdb/cards/KEGG_CALCIUM_SIGNALING_PATHWAY	137	1.99E-04	DOWN
KEGG_RIBOSOME	http://software.broadinstitute.org/gsea/msigdb/cards/KEGG_RIBOSOME	84	7.14E-04	UP
KEGG_ALDOSTERONE_REGULATED_SODIUM_REABSORPTION	http://software.broadinstitute.org/gsea/msigdb/cards/KEGG_ALDOSTERONE_REGULATED_SODIUM_REABSORPTION	35	0.002092255	DOWN
KEGG_VASCULAR_SMOOTH_MUSCLE_CONTRACTION	http://software.broadinstitute.org/gsea/msigdb/cards/KEGG_VASCULAR_SMOOTH_MUSCLE_CONTRACTION	92	0.011150197	DOWN
KEGG_AMINOACYL_TRNA_BIOSYNTHESIS	http://software.broadinstitute.org/gsea/msigdb/cards/KEGG_AMINOACYL_TRNA_BIOSYNTHESIS	41	0.015030571	UP
KEGG_NATURAL_KILLER_CELL_MEDIATED_CYTOTOXICITY	http://software.broadinstitute.org/gsea/msigdb/cards/KEGG_NATURAL_KILLER_CELL_MEDIATED_CYTOTOXICITY	99	0.016282996	DOWN
KEGG_ARRHYTHMOGENIC_RIGHT_VENTRICULAR_CARDIOMYOPATHY_ARVC	http://software.broadinstitute.org/gsea/msigdb/cards/KEGG_ARRHYTHMOGENIC_RIGHT_VENTRICULAR_CARDIOMYOPATHY_AI	63	0.019583456	DOWN

qValue negative log fold change

geneset	URL	size	qValue	direction
KEGG_ALZHEIMERS_DISEASE	http://software.broadinstitute.org/gsea/msigdb/cards/KEGG_ALZHEIMERS_DISEASE	141	0	DOWN
KEGG_CELL_CYCLE	http://software.broadinstitute.org/gsea/msigdb/cards/KEGG_CELL_CYCLE	121	0	DOWN
KEGG_DNA_REPLICATION	http://software.broadinstitute.org/gsea/msigdb/cards/KEGG_DNA_REPLICATION	36	0	DOWN
KEGG_HUNTINGTONS_DISEASE	http://software.broadinstitute.org/gsea/msigdb/cards/KEGG_HUNTINGTONS_DISEASE	155	0	DOWN
KEGG_OOCYTE_MEIOSIS	http://software.broadinstitute.org/gsea/msigdb/cards/KEGG_OOCYTE_MEIOSIS	100	0	DOWN
KEGG_PARKINSONS_DISEASE	http://software.broadinstitute.org/gsea/msigdb/cards/KEGG_PARKINSONS_DISEASE	106	0	DOWN
KEGG_PATHOGENIC_ESCHERICHIA_COLI_INFECTION	http://software.broadinstitute.org/gsea/msigdb/cards/KEGG_PATHOGENIC_ESCHERICHIA_COLI_INFECTION	53	0	DOWN
KEGG_PROTEASOME	http://software.broadinstitute.org/gsea/msigdb/cards/KEGG_PROTEASOME	41	0	DOWN
KEGG_STEROID_BIOSYNTHESIS	http://software.broadinstitute.org/gsea/msigdb/cards/KEGG_STEROID_BIOSYNTHESIS	16	0	DOWN
KEGG_SYSTEMIC_LUPUS_ERYTHEMATOSUS	http://software.broadinstitute.org/gsea/msigdb/cards/KEGG_SYSTEMIC_LUPUS_ERYTHEMATOSUS	112	0	DOWN
KEGG_REGULATION_OF_ACTIN_CYTOSKELETON	http://software.broadinstitute.org/gsea/msigdb/cards/KEGG_REGULATION_OF_ACTIN_CYTOSKELETON	184	3.79E-04	DOWN
KEGG_P53_SIGNALING_PATHWAY	http://software.broadinstitute.org/gsea/msigdb/cards/KEGG_P53_SIGNALING_PATHWAY	66	5.83E-04	DOWN
KEGG_PATHWAYS_IN_CANCER	http://software.broadinstitute.org/gsea/msigdb/cards/KEGG_PATHWAYS_IN_CANCER	293	0.006809834	DOWN
